# Data Mining and Machine Learning Models for Predicting Drug Likeness and Their Disease or Organ Category

**DOI:** 10.3389/fchem.2018.00162

**Published:** 2018-05-09

**Authors:** Abraham Yosipof, Rita C. Guedes, Alfonso T. García-Sosa

**Affiliations:** ^1^Department of Information Systems and Department of Business Administration, College of Law & Business, Ramat-Gan, Israel; ^2^Department of Medicinal Chemistry, Faculty of Pharmacy, Research Institute for Medicines (iMed.ULisboa), Universidade de Lisboa, Lisbon, Portugal; ^3^Department of Molecular Technology, Institute of Chemistry, University of Tartu, Tartu, Estonia

**Keywords:** machine-learning, drug, data-mining, logistic, organ, drug design, multi-target

## Abstract

Data mining approaches can uncover underlying patterns in chemical and pharmacological property space decisive for drug discovery and development. Two of the most common approaches are visualization and machine learning methods. Visualization methods use dimensionality reduction techniques in order to reduce multi-dimension data into 2D or 3D representations with a minimal loss of information. Machine learning attempts to find correlations between specific activities or classifications for a set of compounds and their features by means of recurring mathematical models. Both models take advantage of the different and deep relationships that can exist between features of compounds, and helpfully provide classification of compounds based on such features or in case of visualization methods uncover underlying patterns in the feature space. Drug-likeness has been studied from several viewpoints, but here we provide the first implementation in chemoinformatics of the t-Distributed Stochastic Neighbor Embedding (t-SNE) method for the visualization and the representation of chemical space, and the use of different machine learning methods separately and together to form a new ensemble learning method called AL Boost. The models obtained from AL Boost synergistically combine decision tree, random forests (RF), support vector machine (SVM), artificial neural network (ANN), *k* nearest neighbors (kNN), and logistic regression models. In this work, we show that together they form a predictive model that not only improves the predictive force but also decreases bias. This resulted in a corrected classification rate of over 0.81, as well as higher sensitivity and specificity rates for the models. In addition, separation and good models were also achieved for disease categories such as antineoplastic compounds and nervous system diseases, among others. Such models can be used to guide decision on the feature landscape of compounds and their likeness to either drugs or other characteristics, such as specific or multiple disease-category(ies) or organ(s) of action of a molecule.

## Introduction

An important task in drug design is to guide the synthesis, purchase, and testing of compounds based on their predicted properties. Proper prediction of properties can save time and resources, but also generate compounds not available beforehand. There are several methods to compare a real or virtual compound to known collection of compounds, from topological similarity, fingerprints, molecular features, among others (Ivanenkov et al., 2009; Akella and DeCaprio, 2010; García-Sosa et al., 2010, 2012a,b; Dhanda et al., 2013).

Machine learning allows observing hidden patterns in data, and modifying algorithms in order to better discern the patterns and improve robustness (Schneider, 2017; Gómez-Bombarelli et al., 2018). This includes several layers of data (deepness) and optimization of a function in order to better adopt the features of the (chemical) data (Schneider, 2017; Gómez-Bombarelli et al., 2018). Feedback loops can improve the learning process. The use of artificial intelligence and machine learning can enable the automated design of compounds according to several properties to be optimized (Schneider, 2017; Gómez-Bombarelli et al., 2018).

Visualization of high dimensional data is an important problem in many different domains and especially in drug design. Visualization of chemical data and a good representation of the chemical space are useful in many chemoinformatics and drug design applications including the selection of compounds for synthesis, the selection of compounds for biological evaluation, and the selection of subsets for the design of information-rich compound libraries (Ivanenkov et al., 2009; Akella and DeCaprio, 2010). The main problem of visualization of high dimensional data concerns the data representation in 2D or 3D with minimal loss of information. The dimensionality reduction aim is to preserve as much of the significant structure of the high-dimensional data as possible in the low-dimensional map.

The traditional approach for dimensionality reduction is principal component analysis (PCA) (Jolliffe, 2002), which assumes linear correlation between the dimensions and therefore, cannot adequately handle complex nonlinear data. In the last decade, a number of nonlinear techniques for dimensionality reduction have been proposed and implemented in chemoinformatics, such as self-organized map (SOM) (Zupan and Gasteiger, 1999) and generative topographic map (GTM) (Kireeva et al., 2012), to name but a few. In contrast to traditional linear techniques, nonlinear techniques have the ability to deal with complex nonlinear data, which is pervasive in drug design.

An important factor to consider in machine learning and artificial intelligence, as with any modeling work, is to properly account for the underlying data. The initial and sequential datasets must be well curated, to guarantee that the features and numbers are not biased and that they represent an important classification, optimization, or design task (Schneider, 2017).

Drug design requires an extremely high degree of selectivity. This implies a specific profile of interaction of a compound (or several compounds) with several targets, such as is the case seen in clinic-approved kinase inhibitors, while at the same time avoiding off- or anti-targets that may be responsible for side-effects (Campillos et al., 2008). Disease or organ classification is also important given that the same targets can be present in different tissues and therapeutic compounds need to have an efficient concentration at a specific place for effective action in an organism. These challenges have been approached using probability density functions (García-Sosa et al., 2012a), multivariate logistic regressions (García-Sosa et al., 2012b), PCA (García-Sosa et al., 2012c), and Bayesian naïve classifiers (García-Sosa and Maran, 2013), among others.

In the present work, the t-Distributed Stochastic Neighbor Embedding (t-SNE) method for the visualization and the representation of chemical space is implemented for the first time, and the use of different machine learning methods from decision tree, random forests (RF), support vector machine (SVM), artificial neural network (ANN), k-nearest neighbors (k-NN), and logistic regression models, separately and together, to form a new ensemble learning method called AL Boost for separation of drugs and nondrugs. Good models can also be achieved for disease categories such as antineoplastic compounds, cardiovascular system drugs, and nervous system diseases.

## Methods

### Data set

The full data set contains 762 compounds; compounds were classified into two classes: drug (366 compounds) and non-drug (396 compounds). The compounds were obtained from previous work (García-Sosa et al., 2010, 2012b), where the DrugBank (Wishart et al., 2006) was used to ascertain approved-drug status. Curation included that structure files were checked for consistency (chemical structure corresponds to chemical name) and cleaned, such as removing salts, counterions, etc. All compounds and features are provided in Table S1 on the Supporting Information.

### Properties calculation

Thirty five molecular properties were chosen and calculated for each compound, using ChemAxon^1^ and XLogP (Wang et al., 2000) software, the same properties as in previous publications (see more details on the selection of properties in García-Sosa et al., 2012b; García-Sosa and Maran, 2013).

These physicochemical features were: the binding free energy to their target, Δ*G*_bind_; log*P*; exact mass; Number of Carbons (NoC); Wiener index; molecular surface area (MSA); polar surface area (PSA); apolar surface area (apolarSA); hydrogen bond donor count; hydrogen bond acceptor count; rotatable bond count; atom count; hydrogen count; number of heavy atoms (NHA); molecular polarizability; aliphatic ring count; aromatic ring count; aromatic atom count; Balaban index; Harary index; bond count; hyperWiener index; Platt index; Randic index; ring count; Szeged index; Wiener polarity; and the ligand efficiencies (Kuntz et al., 1999) Δ*G*_bind__NHA; Δ*G*_bind__MW; Δ*G*_bind__PSA; Δ*G*_bind__MSA; Δ*G*_bind__apolarSA; Δ*G*_bind__Wiener; Δ*G*_bind__*P*; Δ*G*_bind__NoC. Free energies of binding were calculated using inhibition or dissociation constants from the SCORPIO (Ababou and Ladbury, 2007), KiBank (Zhang et al., 2004), and PDBbind (Wang et al., 2004) databases. Non-drugs had their nonexistence as drugs verified in the DrugBank (Wishart et al., 2006). Together, they composed a balanced set of drugs and nondrugs, which is important in order not to bias or skew the feature patterns toward one group of compounds against the other. Important features of the sets are that their distribution of binding energies and the number of compounds is similar for both drugs and non-drugs, and that the drugs include all administration routes, not only oral. This introduces a challenge to distinguish drugs from active, non-therapeutic compounds (non-drugs) because the differences between drugs and non-drugs are not judged by their binding energy (i.e., not only potency determines drug likeness), since other features then become more important to distinguish these groups of compounds.

In order to make a comparison to previous studies, no properties selection has been done, although to initially evaluate these properties, an information gain procedure has been implemented. Briefly, the information gain (Mitchell, 1997) of a property reflects the “degree of purity” of the partition obtained by splitting the parent data set using this property. The degree of purity is determined according to Shannon's entropy measure. This method has been widely used in chemoinformatics and bioinformatics and in a recent comparative study it was found to be highly effective for properties selection prior to model generation (Liu, 2004; Saeys et al., 2007). The information gain results indicated information gain (greater than 0) for 30 properties and no information gain (equal to zero) for five properties namely: hydrogen count; Platt index; ring count; Balaban index, and Δ*G*_bind__NoC.

The models and visualization use normalization of the features as a standard procedure.

### Data mining workflow

The data mining procedure is derived using a workflow consisting of two main stages as follows: (1) Data visualization using the t-SNE method; (2) Classification models, which starts by dividing the data into training and test sets, followed by model generation with seven classification methods and model validation.

### Data visualization

The t-Distributed Stochastic Neighbor Embedding (t-SNE) (Maaten and Hinton, 2008) method is a non-linear dimensionality reduction algorithm that is especially designed for embedding high-dimensional data into a space of 2D or 3D. The t-SNE is capable of capturing much of the local information of the high-dimensional data, while also revealing global information such as clusters in the low dimensional representation. The basic idea of t-SNE is that similar objects are modeled by nearby points and dissimilar objects are modeled by distant points in the low dimensional embedding. The t-SNE algorithm consists of three main stages:

1. Collection of the pairwise Euclidean distances between all high dimensional objects and convert them into conditional probabilities and then into joint probabilities, where similar objects get high probability and dissimilar ones get small probability
2. Creation of an initial set of low-dimensional objects
3. Iteratively update the low-dimensional objects to minimize a fitness function (the Kullback-Leibler (KL) divergence, i.e., how one probability distribution diverges from a second expected probability distribution) between a Gaussian distribution in the high-dimensional space and a *t* distribution in the low-dimensional space.

In order to evaluate the ability of the low dimensional representation to preserve the high-dimensional data and structure, we used the trust measure (Venna and Kaski, 2006). The trust measure defines the millieu of compounds, so that the neighborhood in the low dimensional representation is similar to the high dimension, and is given by Equation (1):

(1) $$\begin{array}{r} Trust=\frac{1}{n}*\frac{1}{k}\overset{n}{\underset{i\;=\;1}{\sum }}\overset{k}{\underset{j\;=\;1}{\sum }}\delta_{j}\left(s_{i,j},\bar{x_{i}}\;\right) \end{array}$$

where *n* represents the number of compounds, *k* the number of nearest neighbors, *s*_*i, j*_ represents neighbor *j* for compound *i* in the low dimension representation, and $\bar{x_{i}}\;$is the vector of compound *i* neighbors in the high dimension. δ_*j*_ is defined to be 1 if *s*_*i, j*_ is found in $\bar{x_{i}}$ or 0 if not.

In this work, the neighborhood is defined as the 10 nearest neighbors, and we used the t-SNE algorithm as implemented in the MATLAB version R2017b.

### Classification models-selection of training set and test set

In order to validate the classification model, compounds were divided into a training set (80%, 610 compounds), and a test set (20%, 152 compounds). Similar proportions (20%) of drug (73 compounds) and non-drug (79 compounds) compounds were selected for the test sets by applying independent selection procedures of a representativeness function (Yosipof and Senderowitz, 2014) to the two activity categories. Briefly, this method uses a simulated annealing optimization to select a subset of objects (e.g., compounds) which best represents the parent database from which it was selected. The models were built on the training set by using 10-fold cross-validation and seven methods and then tested on the test set.

### Classification methods

Six different algorithms, namely, decision tree, random forests (RF), support vector machine (SVM), artificial neural network (ANN), *k*-nearest neighbors (*k-*NN) and logistic regression (LR), and one newly boosting method named AL Boost, were used to build the classification models. In each case, a classification model was built using the training set and subsequently used to predict the activities (drug status) of the test set compounds for validation. The six models were generated with algorithms implemented in the WEKA version 3.9.1 (Hall et al., 2009) software using default parameters unless otherwise noted and the newly boosting method was self-coded.

The decision tree algorithm (Quinlan, 1986) operates by iteratively splitting a dataset characterized by activity data and features into smaller subsets. At each step, all features are considered in the search for one that, upon splitting a parent node, would produce the most uniform (activity-wise) child nodes. This procedure is repeated until no more splits are warranted, since either all compounds within all (terminal) nodes have identical activities, or since the gain in uniformity upon additional splits is not statistically significant. In the present study, we used the J4.8, a C4 variant algorithm.

Random forests (RF) (Breiman, 2001), as developed in 2001, with Breiman introducing the principle of random forests as an extension to the decision tree algorithm. In RF, multiple trees (rather than a single tree) are generated using randomly selected feature sets. Activity predictions are made by all trees and combined using a majority vote rule. In the present study, the number of trees was set to the default value of 100.

Support vector machine (SVM) (Vapnik, 1995) is an algorithm which has proven useful for noisy data. Under this paradigm, models are built by identifying a rigid decision hyperplane which leads to the greatest possible margins between activity classes. Nonlinear data could be handled by transposing the original feature space to higher dimensionalities using kernels. In this study, we have chosen to use the polynomial kernel function.

Artificial neural network (ANN) (Hassoun, 1995) is a non-linear classification method inspired by the behavior of biological networks of neurons. Within this approach, objects (i.e., compounds) are represented by vectors containing their features. Each feature is passed to one of the input neurons to which a weight is assigned. Based on these weights, input is passed to the output layer over a number of (optional) hidden layers. The output layer combines these signals to produce a result (e.g., activity or class prediction). Initially, weights are set to random values. As the network is repeatedly presented with input data, these weights are adjusted so that the total output of the network approximates the observed endpoint values associated with the compounds. In the present study we used multilayer perceptrons (MLP) with 19 hidden layers and 19 nodes.

*k*-Nearest Neighbor (*k-*NN) (Mitchell, 1997) is a *lazy learning* classification method, which assigns new compounds to the most common class of known compounds in their immediate neighborhood. Closest neighbors are identified by calculating Euclidian distances in a pre-defined feature space. In this present study, we used *k* = 5 neighbors.

The logistic regression (LR) (Mitchell, 1997) is a type of regression analysis where the dependent variable is binary (or binomial). The model is simply a non-linear transformation of the linear regression. The result is an equation which includes the impact of each variable on the odds ratio of the observed event of interest.

AL Boost: is a new chemoinformatics ensemble learning classification method which combines all the models obtained in this work (i.e., J4.8, RF, SVM, ANN, *k-*NN, and LR) together into one predictive model in order to improve the predictive force and decrease the bias. This method takes the predictions of each classification (learners) and combines them using a weighted majority voting function to determine the prediction of each compound. Each learner is assigned a weight according to its corrected classification rate error, given that poor learners get lower weights. For each compound, two functions are calculated as follows:

(2) $$\begin{array}{r} f\left(active\right)=\overset{n}{\underset{i\;=\;1}{\sum }}\frac{1}{w_{i}}*\delta_{i} \end{array}$$

(3) $$\begin{array}{r} f\left(inactive\right)=\overset{n}{\underset{i\;=\;1}{\sum }}\frac{1}{w_{i}}*\delta_{i} \end{array}$$

where *i* are the learner methods, *w*_*i*_ is the corrected classification rate error (CCR error, Equation 4) of learner *i*, and δ_*i*_ is 1 if the learner is predicted as active class (e.g., drug), or 0 if predicted as inactive class (e.g., non-drug), for Equation (2).

For Equation (3), δ_*i*_ is 1 if the learner is predicted as inactive class (e.g., non-drug) or 0 if it is predicted as active class (e.g., drug).

The majority vote between Equations (2) and (3) determines the prediction for the compound.

The last classification method detailed in this paper is Naïve Bayesian classifiers. This method was used in a previous publication (García-Sosa and Maran, 2013), thus it was not used for model building in this study, rather for comparison to the results obtained in García-Sosa and Maran (García-Sosa and Maran, 2013). The Naïve Bayesian classifiers use the distributions of features for different classes, and construct Gaussians for describing these distributions with characteristics being the mean and standard deviation. The probabilities (*P*) of a compound with certain features belonging to either class are computed, and that compound is assigned to the class for which the highest *P* is obtained.

### Classification models-prediction statistics

In all cases, classification predictions were evaluated using the corrected classification rate (CCR, also called “balanced accuracy”), accuracy, Matthews correlation coefficient (MCC), sensitivity, specificity, and the variance between the sensitivity and the specificity (Equations 5–10), where sensitivity is the percentage of truly active (e.g., drug) compounds being predicted from the model (Equation 8), and specificity is the percentage of truly inactive (e.g., non-drug) compounds being predicted from the model (Equation 9).

(4) $$\begin{array}{r} CCR\text{ error}=1-\frac{1}{2}\left(\frac{T_{N}}{N_{N}}+\frac{T_{P}}{N_{P}}\right) \end{array}$$

(5) $$\begin{array}{r} CCR=\frac{1}{2}\left(\frac{T_{N}}{N_{N}}+\frac{T_{P}}{N_{P}}\right) \end{array}$$

(6) $$\begin{array}{r} Accuracy=\frac{T_{N}+T_{P}}{{N_{N}+N}_{P}} \end{array}$$

(7) $$\begin{array}{r} MCC=\frac{T_{N}T_{P}-F_{N}F_{P}}{\sqrt{\left(T_{P}+F_{P}\right)\left(T_{P}+F_{N}\right)\left(T_{N}+F_{P}\right)\left(T_{N}+F_{N}\right)}\;} \end{array}$$

(8) $$\begin{array}{r} Sensitivity=\frac{T_{P}}{T_{P}+F_{N}} \end{array}$$

(9) $$\begin{array}{r} Specificity=\frac{T_{N}}{T_{N}+F_{P}} \end{array}$$

(10) $$\begin{array}{r} Variance=\frac{1}{2}*{\left(Sensitivity-\mu \right)}^{2}+\frac{1}{2}*{\left(Specificity-\mu \right)}^{2} \end{array}$$

where *T*_*N*_ and *T*_*P*_ represent the number of true negative (e.g., non-drug) and true positive (e.g., drug) predictions, respectively. *N*_*N*_ and *N*_*P*_ represent the total number of the two activity classes, and *F*_*N*_ and *F*_*P*_ represent the number of false negative and false positive predictions, respectively. μ represents the mean of the sensitivity and specificity.

### Disease categories

Further in the analysis of the drug/non-drug database, the drugs in the data set were characterized into their different anatomic therapeutical classifications, also called disease or organ category (DC). Here the comparison was not drugs *vs*. non-drugs, but drugs of one DC against another DC. In this work, we focus on the three largest DCs, namely cardiovascular, anti-neoplastic, and nervous systems. These three groups were evaluated against each other preforming three sub data sets Cardiovascular drugs vs. Anti-neoplastic agents, Cardiovascular drugs vs. Nervous system, and Anti-neoplastic agents vs. Nervous system. The same data mining workflow as before was applied. The number of compounds and the number of training and test sets (similar procedure and proportions as in the drug/non-drug database) for each DC are presented in Table 1.

**Table 1:** The number of compounds, training, and test sets for the three different disease/organ category.

**Disease/organ category**	**Compounds**	**Training set**	**Test set**
Cardiovascular drugs	56	44	12
Anti-neoplastic agents	20	16	4
Nervous system	111	89	22

## Results and discussion

The properties of the compounds include widely used metrics such as size, weight, polarity, as well as topological indices, and ligand efficiencies. An important consideration for the data set construction was the curation of binding free energies of similar magnitude between drugs and non-drugs (bioactive compounds). Ligand efficiencies can normalize the binding energy of a compound according to other properties of a compound, such as size, lipophilicity, etc., and have a pragmatic use in developing series of compounds in order to improve or maintain binding strength while also improving their profile in other dimensions.

**Figure 1:**
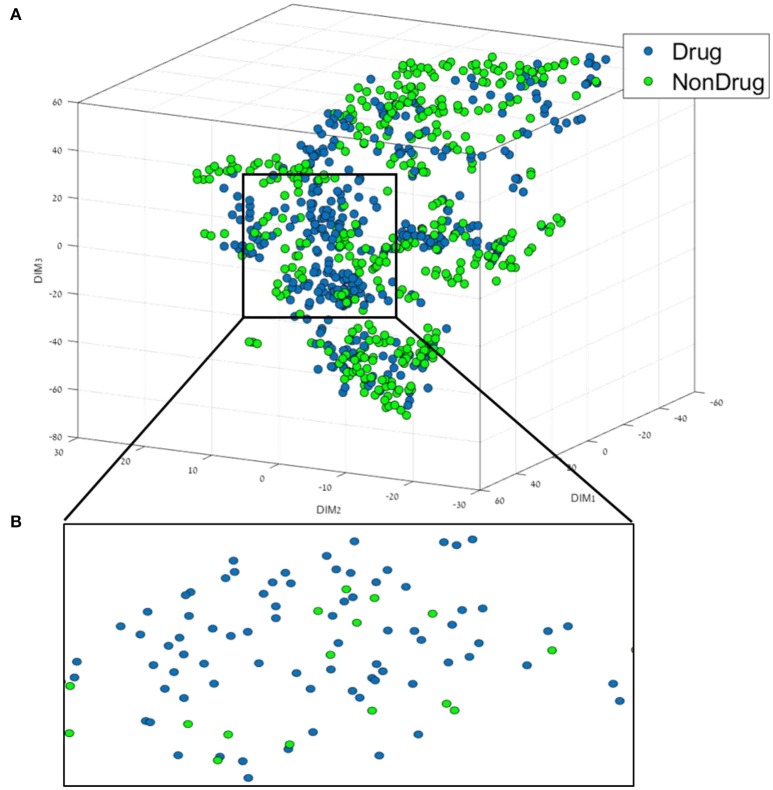
**(A)** 3D representation of the Drug/Non-drug feature space using the t-SNE algorithm for dimension reduction. **(B)** A zoom into the drug area from the black square in **(A)**.

The first step of the data mining workflow is data visualization; the resulting 35 dimensional data of the drug/non-drug database was reduced into a 3D representation using t-SNE. The resulting trust measure of the low dimensional embedding was found to be 63%. A comparison to the common dimension reduction technique PCA, found that for a 3D representation using PCA, a trust measure of only 42% was obtained. This result indicates a good preservation of the structure and of the local information of the high dimensional data in the low embedding for the t-SNE. The distribution and the chemical space of the drug/non-drug compounds in the resulting t-SNE 3D space are presented in Figure 1.

From Figure 1, it can be broadly seen that drug compounds occupy mostly the central area (shown in the black square in Figure 1) of the plot in this perspective plane, and non-drugs are among the edges. The second step of the data mining workflow is the building and validation of the classification models. In order to build the classification models, we used six different classification methods, as well as the boosting method (AL Boost), the results obtained are shown in Table 2.

**Table 2:** Drug/non-drug classification results.

	**Training set**						**Test set**					
**Model**	**Specificity**	**Sensitivity**	**CCR**	**Accuracy**	**Variance**	**MCC**	**Specificity**	**Sensitivity**	**CCR**	**Accuracy**	**Variance**	**MCC**
J4.8	0.67	0.67	0.67	0.67	0.01	0.35	0.72	0.81	0.75	0.76	18.90	0.52
RF	0.76	0.76	0.76	0.76	0.00	0.52	0.80	0.86	0.82	0.82	10.21	0.65
*k-*NN	0.73	0.73	0.73	0.73	0.06	0.46	0.75	0.78	0.76	0.76	1.34	0.53
SVM	0.73	0.74	0.73	0.73	0.25	0.47	0.74	0.73	0.74	0.74	0.17	0.47
ANN	0.77	0.72	0.75	0.75	5.25	0.50	0.81	0.81	0.81	0.81	0.12	0.62
LR	0.76	0.76	0.76	0.76	0.01	0.52	0.77	0.70	0.74	0.74	12.58	0.48
AL Boost	0.76	0.77	0.76	0.76	0.01	0.53	0.80	0.81	0.81	0.81	0.22	0.62
Naïve Bayesian^*^	0.74	0.89	–	0.82	–	0.64	0.50	0.92	–	0.70	–	0.46

* *Naïve Bayesian results were taken from García-Sosa and Maran (2013)*.

These results show that the separation between classes is good, and comparable to those found in other studies (García-Sosa and Maran, 2013). The AL Boost method performed as well as the best of the individual methods. The overall performances of the different models for the training set were evaluated by the CCR and are between 0.67 and 0.76. The best classification models based on this criterion are the AL Boost, the RF, and the LR (with CCR = 0.76) followed by the ANN (CCR = 0.75), SVM and k-NN (with CCR = 0.73), while the decision tree (with CCR = 0.67) lags behind. The overall performances of the different models for the test set largely mirror the results from the training set. However, some methods performed better, and the AL Boost method gave a good CCR of 0.81, while random forest had the highest CCR value of 0.82. On average, the CCR of the six other methods is 0.73 and 0.77 for the training set and test set, respectively, which is lower than the CCR results for AL Boost, but not statistically significant.

In order to evaluate the new AL Boost method, the variance between the specificity and the sensitivity was calculated as well. The variance represents the model balance between the two classes or the bias of the model toward one class. Lower variance values represent low bias and balanced models while higher variance values represent high bias and unbalanced models. The results for the training set and test set for the AL Boost method represent very similar results of specificity and sensitivity (training set 0.76 and 0.77 and test set 0.80 and 0.81 for the specificity and sensitivity, respectively). These results show a low bias and a well-balanced model with a variance of 0.01%^2^ and 0.22%^2^ for the training and test set, respectively, while on average, the variance of the six other methods is 0.93%^2^ and 7.22%^2^ for the training and test set, respectively. In addition, comparing the AL Boost to our previous publication (García-Sosa and Maran, 2013) using the naïve Bayesian method shows that the accuracy of AL Boost (0.81) is higher on the test set than the Bayesian classifier (0.70).

To evaluate the features used in this research, Table 3 represents the most frequent features selected by the classifiers for the final model. In this case, the most frequent features are those that were both selected by the decision tree model and by the logistic regression models, while the other methods used all the features or combination of them for the final model. A comparison to previous publications reveals that there is common ground: the features of Acceptor Count, Donor Count, PSA, Log*P*, Δ*G*_bind__MSA, and Balaban Index were found to be features that separated drug and nondrug in REF (García-Sosa and Maran, 2013) and in REF (García-Sosa et al., 2012b). In addition, the Balaban Index was found to have an information gain of zero in the initial evaluation of the features (see Methods section), but here it was selected as one of the features that can separate drug and nondrug. Furthermore, it is interesting to note that most of the properties that correspond to the ones in Lipinski's rule of five (Lipinski et al., 2001) were found to have the ability to split the data set into drugs and non-drugs classes.

**Table 3:** Most frequent model selected features.

**Features**		
Acceptor count	Δ*G*_*bind*_	Δ*G*_*bind*__PSA
Donor count	Rotatable bond count	Δ*G*_bind__MSA
PSA	Hydrogen count	Δ*G*_bind__*P*
LogP	Aliphatic ring count	Number of carbons
Aromatic ring count	Balaban index	

**Figure 2:**
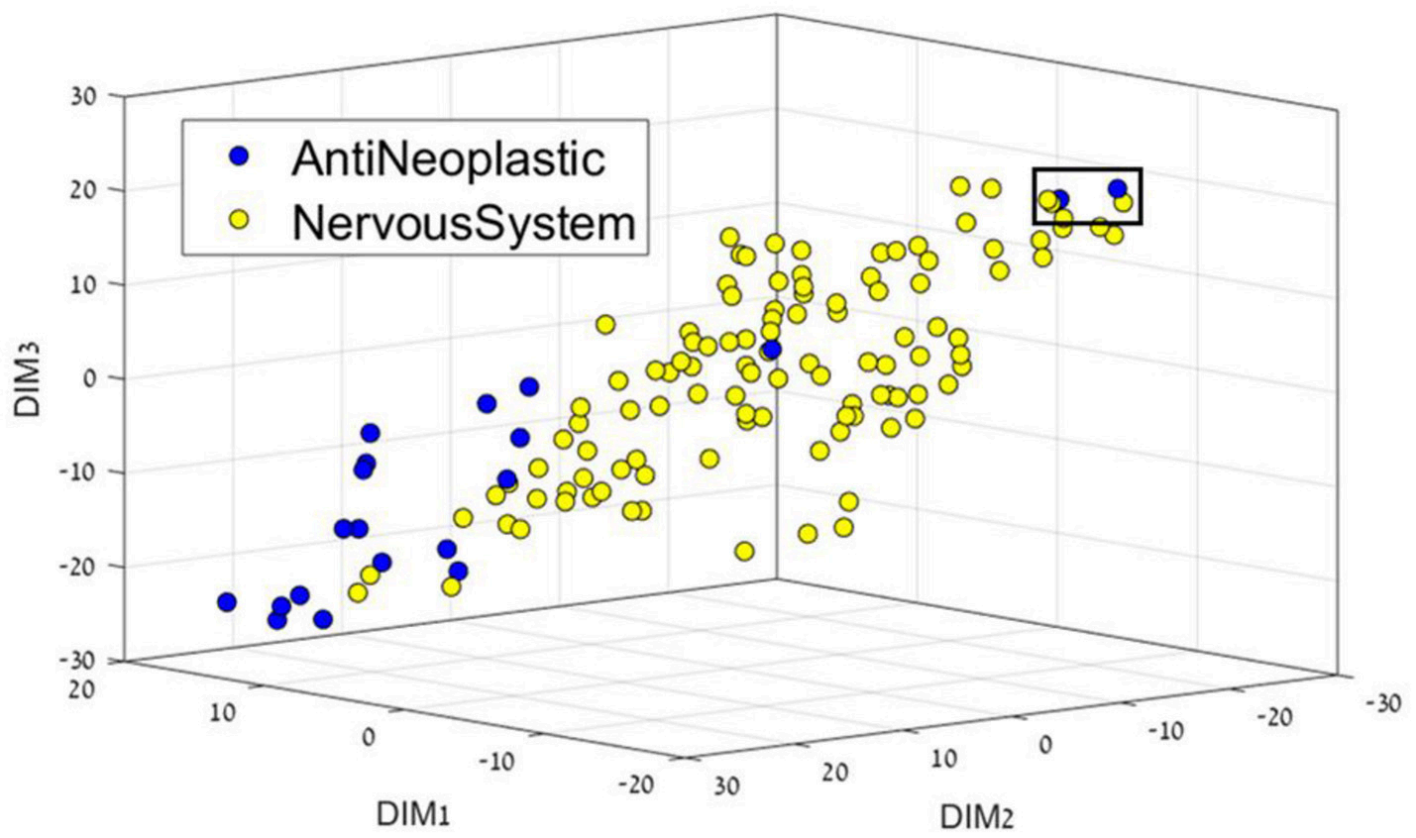
3D representation of the Anti-Neoplastic-Nervous system feature space using the t-SNE algorithm for dimension reduction. Two Anti-Neoplastic compounds (two blue dots in the black square) are markedly different from the rest of the Anti-neoplastic bulk. They correspond to compounds fluorouracil (5-FU), an atypically small compound – one ring –, and to pentostatin, also a small, polar compound; both of them acting as nucleoside analogs.

**Figure 3:**
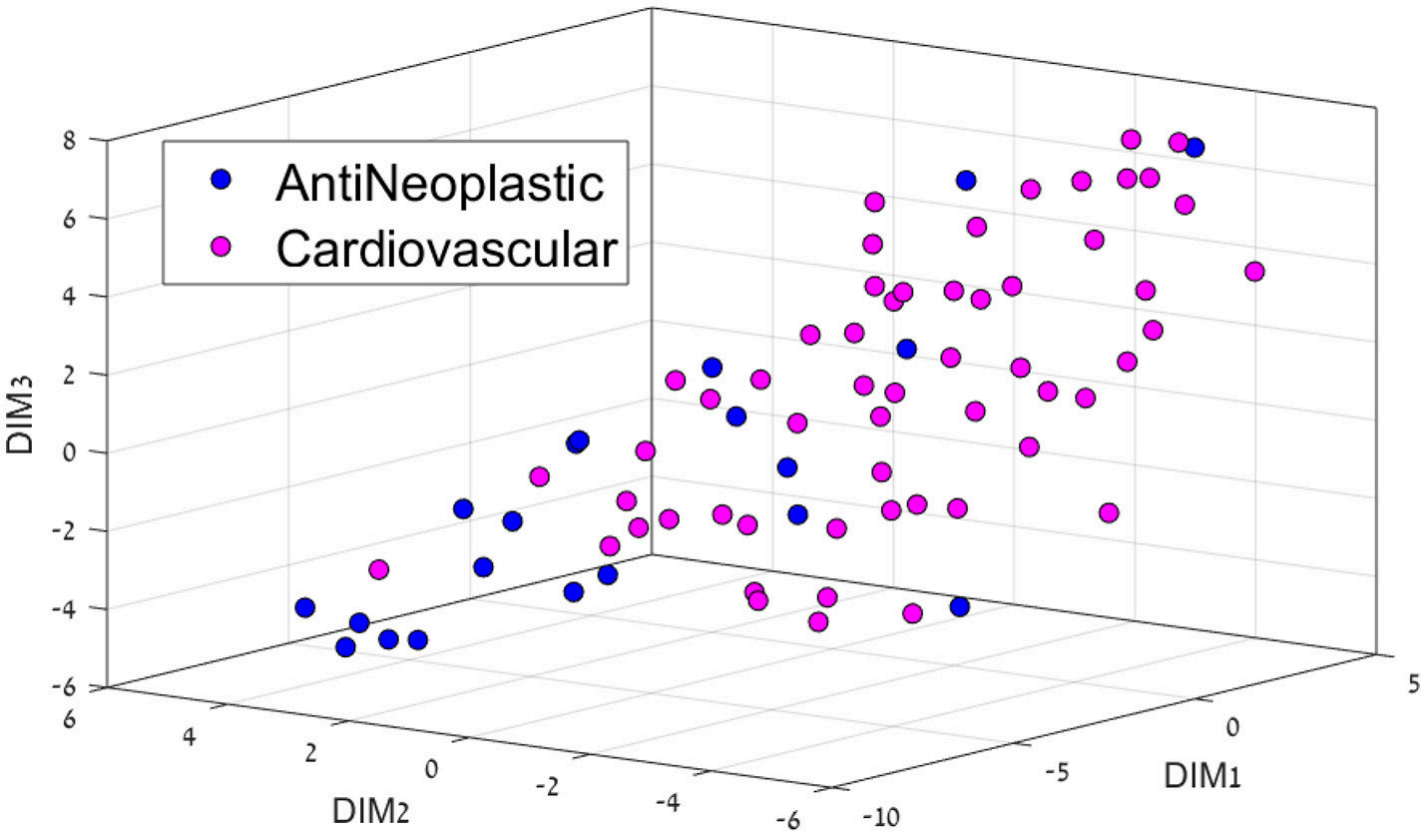
3D representation of the Anti-Neoplastic-Cardiovascular feature space using the t-SNE algorithm for dimension reduction.

**Figure 4:**
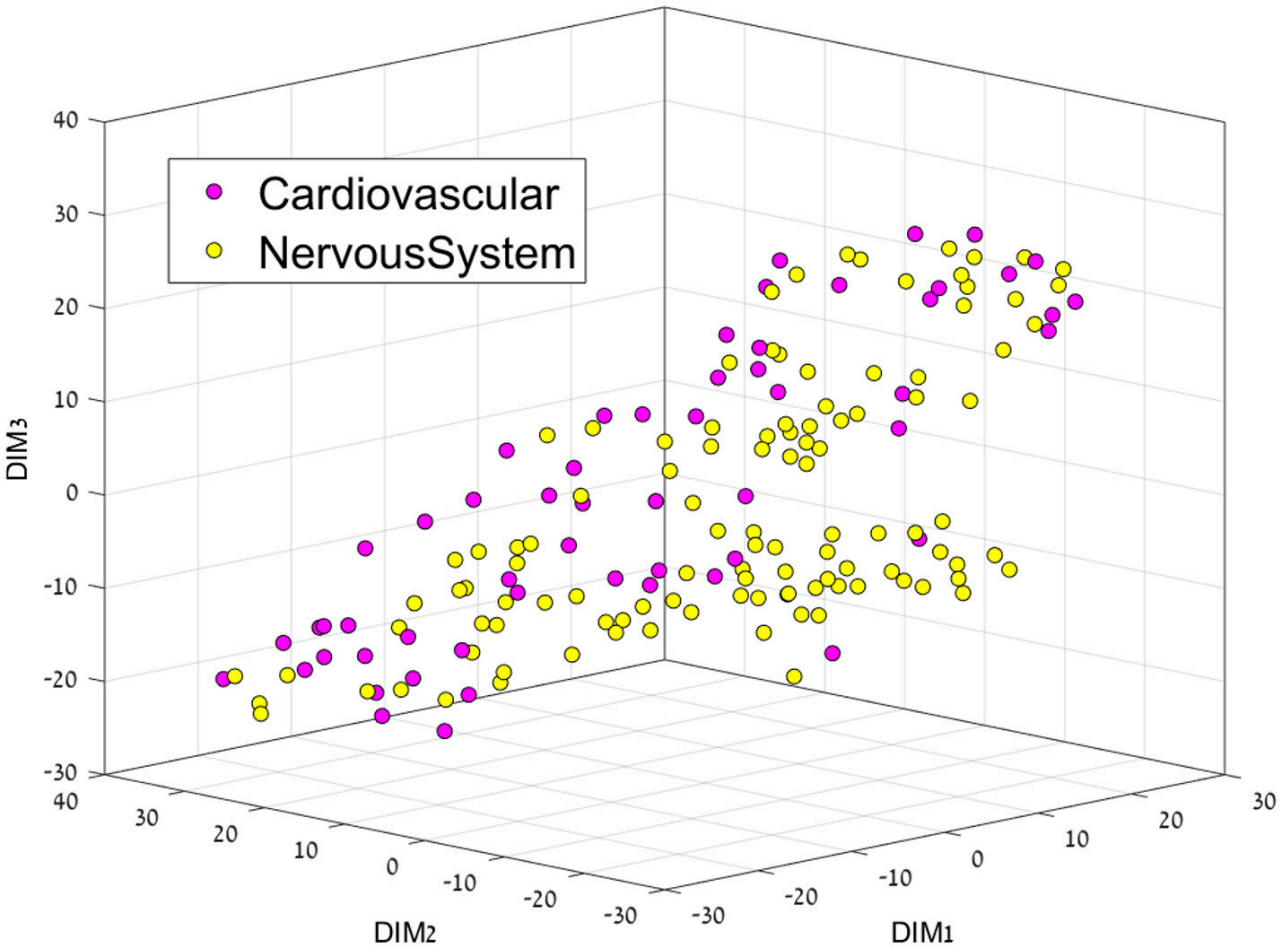
3D representation of the Cardiovascular-Nervous System feature space using the t-SNE algorithm for dimension reduction.

Having separated drugs and non-drugs, the next step was to consider separation of drugs into their different disease or organ category (DC). First, we visualize the feature space using t-SNE for each sub dataset. The resulting 3D representation of Anti-Neoplastic-Nervous system, Anti-Neoplastic-Cardiovascular, and Cardiovascular-Nervous system can be seen in Figures 2–4, respectively. The trust measure results can be seen in Table 4; these results clearly show the ability of the t-SNE methods to preserve the high-dimensional data and structure, with a trust between 70 and 74% for the three datasets. As before, a comparison to PCA was done, and again the t-SNE trust results were found to overcome the PCA. Although the trust results were found to be higher for the t-SNE than the PCA for each data set, no statistically significant difference was found between them.

**Table 4:** The trust results for the low-dimensional embedding (3D representation) using t-SNE and PCA.

**System**	**t-SNE (%)**	**PCA (%)**
Anti-neoplastic vs. nervous	72	66
Anti-neoplastic vs. cardiovascular	74	72
Cardiovascular vs. nervous	70	62

Figure 2 presents the Anti-Neoplastic-Nervous system 3D representation. In this 3D representation a clear separation between the two DCs can be seen, with nervous system drugs in the center, and cancer drugs mostly on the edges, or not as well defined as nervous system. If one considers their place or target of action, nervous system drugs require locating in the brain and CNS, which requires the passing of specific membranes such as the blood-brain barrier that imposes a feature profile in the compounds. Two Anti-Neoplastic compounds (Figure 2, two blue dots in the black square, DR109.mol2, DR211.mol2) are markedly different from the rest of the Anti-neoplastic bulk. They correspond to compounds fluorouracil (5-FU), an atypically small compound – one ring –, and to pentostatin, also a small, polar compound; both of them acting as nucleoside analogs.

Cardiovascular drugs were also majorly separated from cancer drugs (Figure 3), located mostly in the center as opposed to mostly on the edges for cancer drugs. Cancer affects all organs, so these system drugs tend to be not very located, which is also a major problem with cancer treatment, as side-effects are very common.

This separation between groups was not the case for the Cardiovascular vs. Nervous system drug plots, since both groups are very similar, perhaps only a cluster of cardiovascular drugs can be seen on the middle right.

Having visualized the data, the next step is to build classification models using the six classification methods and the ensemble learning AL Boost. The results for the Anti-Neoplastic vs. Nervous system, Anti-neoplastic vs. Cardiovascular, and Cardiovascular vs. Nervous System are presented in Tables 5–**7**, respectively.

**Table 5:** Classification results of anti-neoplastic vs. nervous system drugs, where specificity represents the nervous system and sensitivity represents the anti-neoplastic.

	**Training set**						**Test set**					
**Model**	**Specificity**	**Sensitivity**	**CCR**	**Accuracy**	**Variance**	**MCC**	**Specificity**	**Sensitivity**	**CCR**	**Accuracy**	**Variance**	**MCC**
J4.8	0.90	0.64	0.70	0.88	179.41	0.46	1.00	0.67	0.95	0.92	277.78	0.78
RF	0.93	0.82	0.77	0.91	28.81	0.63	0.96	1.00	0.88	0.96	4.73	0.85
*k-*NN	0.89	0.83	0.65	0.89	7.72	0.47	0.95	0.75	0.85	0.92	104.60	0.70
SVM	0.92	1.00	0.75	0.92	17.00	0.68	0.95	0.75	0.85	0.92	104.60	0.70
ANN	0.93	0.71	0.79	0.90	120.76	0.61	0.96	1.00	0.88	0.96	4.73	0.85
LR	0.92	0.89	0.74	0.91	1.93	0.63	0.96	1.00	0.88	0.96	4.73	0.85
AL Boost	0.92	0.89	0.74	0.91	1.93	0.63	0.96	1.00	0.88	0.96	4.73	0.85
Naïve Bayesian^*^	–	–	–	0.88	–	0.57	–	–	–	0.90	–	0.62

* Naïve Bayesian results were taken from García-Sosa and Maran (2013)

The results in Table 5 for the anti-Neoplastic vs. Nervous system classification models show good results for the training set with CCR ≥ 0.65, as well as for the test set with CCR ≥ 0.85. The ensemble learning model AL Boost provided a CCR of 0.74 and 0.88 on the training and test set, respectively, with the lowest variance of 1.93%^2^ and 4.73%^2^ on the training and test set, respectively. The accuracy for the AL Boost method was high, 0.91 for the training set, and 0.96 for the test set. These values are better when compared to those obtained previously using naïve Bayesian classifiers for the same data sets, 0.88 for training set, and 0.90 for the test set (García-Sosa and Maran, 2013). The sensitivity for the AL Boost method was much higher than using Bayesian classifiers, of 0.89 and 1.00 for training and test set for the former, vs. 0.50 and 0.60, respectively, for the latter. The values obtained for specificity are comparable for both methods, 0.97 and 0.96% for training and test sets for Bayesians, vs. 0.92 and 0.86, respectively, for AL Boost.

For the differences between cardiovascular drugs and cancer drugs (Table 6), good results were obtained for the AL Boost with CCR of 0.76 and 0.88 for the training set and test set, respectively, while on average, the CCR of the six other methods is 0.65 and 0.83 for the training set and test set, respectively. In addition, the accuracy for AL Boost is comparable for training set and higher for test set, with 0.85 and 0.94, respectively, vs. 0.88 and 0.90, respectively, for the Bayesian classifiers in previous work (García-Sosa and Maran, 2013). The sensitivity for the AL Boost method was comparable to Bayesian classifiers, with 0.82 and 1.00 for training and test set for the former, vs. 0.83 and 1.00, respectively, for the latter. The values for specificity are much higher for the AL Boost method than for Bayesians, with 0.27 and 0.43 for training and test sets for Bayesians, vs. 0.86 and 0.92, respectively, for AL Boost.

**Table 6:** Classification results of anti-neoplastic vs. cardiovascular drugs, where specificity represents the cardiovascular drugs and sensitivity represents the anti-neoplastic.

	**Training set**						**Test set**					
**Model**	**Specificity**	**Sensitivity**	**CCR**	**Accuracy**	**Variance**	**MCC**	**Specificity**	**Sensitivity**	**CCR**	**Accuracy**	**Variance**	**MCC**
J4.8	0.81	0.54	0.65	0.75	182.32	0.32	0.92	1.00	0.88	0.94	14.79	0.83
RF	0.83	0.62	0.69	0.78	114.92	0.41	0.92	1.00	0.88	0.94	14.79	0.83
*k-*NN	0.78	0.80	0.61	0.78	0.83	0.36	0.92	1.00	0.88	0.94	14.79	0.83
SVM	0.78	0.80	0.61	0.78	0.83	0.36	0.86	1.00	0.75	0.88	51.02	0.65
ANN	0.84	0.60	0.71	0.78	149.38	0.44	0.92	1.00	0.88	0.94	14.79	0.83
LR	0.80	0.55	0.63	0.75	156.83	0.30	0.86	1.00	0.75	0.88	51.02	0.65
AL Boost	0.86	0.82	0.76	0.85	3.79	0.59	0.92	1.00	0.88	0.94	14.79	0.83
Naïve Bayesian^*^	–	–	–	0.79	–	0.40	–	–	–	0.79	–	0.55

* *Naïve Bayesian results were taken from García-Sosa and Maran (2013)*.

Another advantage of the AL Boost method, is that it could find a good distinction between cardiovascular and nervous system drugs with CCR of 0.68 and 0.72 for the training set and test set, respectively (shown in Table 7), which was not the case with naïve Bayesian classifiers, the latter being based on simple relationships between descriptors.

**Table 7:** Classification results of cardiovascular drugs vs. nervous system drugs, where specificity represents the nervous system and sensitivity represents the cardiovascular drugs.

	**Training set**						**Test set**					
**Model**	**Specificity**	**Sensitivity**	**CCR**	**Accuracy**	**Variance**	**MCC**	**Specificity**	**Sensitivity**	**CCR**	**Accuracy**	**Variance**	**MCC**
J4.8	0.76	0.55	0.64	0.70	105.33	0.30	0.74	0.55	0.64	0.68	93.78	0.28
RF	0.78	0.72	0.69	0.77	7.48	0.44	0.83	0.80	0.79	0.82	2.78	0.60
*k-*NN	0.71	0.57	0.59	0.69	51.02	0.22	0.70	0.57	0.60	0.68	43.74	0.23
SVM	0.77	0.82	0.68	0.77	6.87	0.46	0.75	0.83	0.69	0.76	17.36	0.47
ANN	0.78	0.56	0.67	0.71	120.60	0.33	0.83	0.73	0.77	0.79	24.41	0.54
LR	0.76	0.63	0.66	0.73	47.18	0.35	0.76	0.67	0.68	0.74	21.78	0.39
AL boost	0.77	0.73	0.68	0.76	3.17	0.42	0.79	0.70	0.72	0.76	21.01	0.47

Summary of the classification results for the four databases presented here (drug/nondrug, anti-neoplastic vs. nervous system drugs, anti-neoplastic vs. cardiovascular drugs and cardiovascular drugs vs. nervous system drugs): a total of 24 results obtained for the individual classification models (e.g., J4.8, RF, *k-*NN, SVM, ANN, and LR) with an average CCR of 0.69 and 0.80 for the training set and test set, respectively. While the average CCR results for the four models of the AL Boost are 0.74 and 0.82 for the training set and test set, respectively. An independent sample *t*-test was performed among the individual CCR models results and the AL Boost CCR results which found no statically significant difference for the training set and not for the test set results. In summary, the average results for the AL Boost were found to overcome the average results for the individual classification models but weren't found to be significantly higher.

This research has several possible limitations including the data set, the classification method, and the visualization methods. One limitation is the number of compounds per disease category/organ classification. It would be good to have larger datasets for some disease groups, but we used the drugs that are available. New drugs are definitely needed in several categories, including cardiovascular, nervous system, and antineoplastic agents. Other limitations include the features selected by the models. Some of the classification methods employ all of the features in the data set, as well as combinations of different features in the final step. This makes the extraction of individual features harder.

A possible limitation for the t-SNE algorithm is the lack of an explicit mapping function. This limitation does not allow one to place any new data on an already existing map. In that case, a new map must be rebuilt from scratch and therefore, this method cannot be used as a supervised learning method (e.g., classification) for prediction.

## Conclusions

To the best of our knowledge, this is the first example of the usage of t-SNE for the visualization and representation of the chemical space and the use of different machine learning methods separately and together to form a new ensemble learning method called AL Boost. Clear and good separations were obtained with the dimension reduction and machine learning approaches to distinguish drugs and non-drugs, as well as three major classes of drug compounds. The ability to use such tools for the identification of interesting trends, opens up new opportunities for understanding the factors affecting drugs performances and for designing new drugs. Considerations such as drug likeness and drug target, organ, and/or system class are thus made possible, providing another route for designing specificity into ligands and drugs. Clearly, this research should be conducted in close collaboration with experts in the medicinal/pharmaceutical chemistry field to both provide a chemistry-based explanation to the observed trends, as well as to capitalize on the results. We expect that the tools and methods implemented in this work will further be used in medicinal chemistry and drug design research.

## Author contributions

AY applied the t-SNE and models and AL Boost algorithms, analyzed results, and wrote the manuscript. RG revised the manuscript. AG-S designed the research project, collected and curated the data, analyzed the results, and wrote the manuscript.

### Conflict of interest statement

The authors declare that the research was conducted in the absence of any commercial or financial relationships that could be construed as a potential conflict of interest.

## Footnotes

^1^(v4.8.1, M., In; 2007)
